# Effects of progressive temporal-occlusion training on early anticipation of forehand drop-shot direction in badminton under temporal information constraints

**DOI:** 10.3389/fpsyg.2026.2001694

**Published:** 2026-10-28

**Authors:** Wenkai Gao, Kexin Gao, Xingyu Liu, Fengxin Li

**Affiliations:** 1 School of Physical Education, Inner Mongolia Normal University, Hohhot, China; 2 Department of Health Management, Inner Mongolia Minzu Preschool Education College, Ordos, China

**Keywords:** badminton, early anticipation, forehand drop shot, perceptual training, shot-direction anticipation, temporal occlusion

## Abstract

This study examined whether an 8-week progressive temporal-occlusion training program improves early anticipation of forehand drop-shot direction in badminton. Seventy-eight university students with systematic badminton training experience were randomly assigned to a progressive temporal-occlusion group, a full-information active-control group, or a regular-activity control group (*n* = 26 per group); 72 completed both pretest and posttest. The two video-training groups trained twice weekly for 8 weeks. Anticipation was assessed at four occlusion points: 80 ms before racket–shuttle contact, 40 ms before contact, at contact, and 40 ms after contact. Shot-direction anticipation accuracy was the primary performance measure, and correct-response reaction time (RT) was the secondary measure. Data were analyzed using mixed repeated-measures ANOVA, planned comparisons, and trial-level sensitivity analyses. Accuracy showed a significant group × test-session interaction (*p* < 0.001), whereas the group × test-session × occlusion-point interaction was not significant (*p* = 0.185). Relative to the active-control group, the progressive group showed larger accuracy gains at 40 ms before contact and at contact (Holm-adjusted *p* = 0.048 and 0.009, respectively). For correct-response RT, the three-way interaction was significant (*p* < 0.001), with larger reductions in the progressive group at 80 ms before contact, 40 ms before contact, and at contact (all Holm-adjusted *p* = 0.013). Thus, the progressive temporal-occlusion training program produced additional improvements in selected anticipation measures beyond those obtained with dose-matched full-information video practice. The additional accuracy gains were observed at 40 ms before contact and at contact, whereas additional RT reductions extended from 80 ms before contact to contact. Because the three-way interaction for accuracy was not significant, the apparent temporal specificity of the accuracy gains should be interpreted cautiously.

## 1 Introduction

Badminton is characterized by rapid transitions between attack and defense, leaving receivers little opportunity to wait until the shuttle trajectory is fully specified before initiating movement. Instead, they must infer shot direction from information available during the opponent’s preparation, racket swing, and the brief interval around racket–shuttle contact. Anticipation is a perceptual–cognitive process in which an observer predicts an upcoming action outcome from advance information under conditions of temporal pressure and incomplete information. Successful anticipation therefore depends on the rapid extraction of discriminative action information and its integration with sport-specific experience (Mann et al., 2007; Harris et al., 2022). During a forehand drop shot, information from trunk orientation, the hitting arm, and racket motion becomes available before the shuttle’s flight trajectory is fully revealed. If the receiver waits until the trajectory is obvious, the time available for movement initiation and adjustment is substantially reduced. The ability to anticipate drop-shot direction from information available before and around contact is therefore an important component of badminton-specific perceptual skill. Studies conducted in badminton have likewise shown that useful anticipatory information is available before action completion and that more experienced performers generally show superior anticipation (Wang and Zhang, 2007; Wang and Yin, 2008).

Previous work has primarily examined this early anticipation advantage through expert–novice comparisons and the temporal-occlusion paradigm. Temporal occlusion terminates a video at different points before or after contact, progressively restricting later information and thereby revealing what information can support prediction at different stages of the action. More skilled performers can maintain comparatively high anticipation performance when only pre-contact information is available (Abernethy, 1988; Robertson et al., 2022), and badminton studies likewise show reliable expertise-related differences in anticipation performance (De Waelle et al., 2023), while more recent temporal-occlusion evidence indicates that these expertise advantages can be particularly evident before and around racket–shuttle contact (Wu et al., 2026). These findings indicate that skilled anticipation does not depend solely on post-contact shuttle-flight information, but also on effective use of advance action information. Recent research further suggests that sport anticipation relies on the integration of contextual prior information with current kinematic information, and that the use and processing of these information sources vary with expertise and time pressure (Chen et al., 2024; Wang et al., 2025; Lu et al., 2025; Huang et al., 2026). Behavioral and neural evidence therefore indicates expertise-related differences in information integration and processing. However, such studies mainly describe advantages associated with accumulated sport experience and do not directly establish whether early-information use can be improved through a relatively short period of sport-specific perceptual training.

Temporal occlusion can also be used as a training method rather than solely as an assessment paradigm. The rationale is not simply to shorten the video. Training can begin with relatively complete information so that learners repeatedly map an opponent’s action onto the resulting shot direction, after which video offset is progressively moved earlier to reduce the availability of post-contact shuttle-flight information and require judgments from earlier action information. Meta-analytic and systematic-review evidence indicates that temporal-occlusion and perceptual–cognitive training can improve anticipation and decision-making performance in sport, although the magnitude of training effects depends on stimulus presentation, training dose, and test context (Müller et al., 2024; Song et al., 2025; Zhu et al., 2024). Badminton-specific studies further suggest that short-term badminton training can improve sport-related visual-perceptual performance (Shi et al., 2022), while video-based perceptual-cognitive training can enhance anticipation performance (Wang et al., 2025). Nevertheless, comparisons only with no-training controls cannot separate the specific value of temporal occlusion from practice effects arising from repeated video viewing, repeated judgments, and feedback. It also remains unclear whether training benefits are general across information stages or are more evident when information is constrained before and around contact.

Accordingly, we used a three-group randomized training experiment with a dose-matched full-information video condition as an active control. The primary aim was to determine whether 8 weeks of progressive temporal-occlusion training could produce additional improvements in forehand drop-shot direction anticipation in university students with systematic badminton training experience. We first compared pretest-to-posttest changes between progressive temporal-occlusion training and full-information video training to test whether a program combining progressively earlier occlusion with practice under early information constraints would yield benefits beyond general video exposure, repeated anticipation, and feedback. We then examined performance at four occlusion points—80 ms before contact, 40 ms before contact, at contact, and 40 ms after contact—to characterize when any additional training benefits emerged. We expected both video-training conditions to show practice-related improvements, with the additional benefit of progressive temporal occlusion being more evident before and around contact and smaller once early shuttle-flight information became available.

## 2 Methods

### 2.1 Participants

University students aged 18–25 years were recruited if they had at least 2 years of systematic badminton training and had participated regularly during the preceding 6 months. Participants had no national athlete classification, had not undergone long-term training in a sport school or professional team, had normal or corrected-to-normal vision, and had no limitation that would interfere with video viewing, keypress responses, or badminton participation. An *a priori* sample-size calculation was performed in G*Power 3.1 for the group × test-session interaction in anticipation accuracy, assuming an effect size of *f* = 0.20, α = 0.05, and power (1 − *β*) = 0.80. The required minimum sample was 66. Allowing for approximately 15% non-completion, the target sample was set at 78 participants.

A total of 78 participants were recruited and randomized equally across the three groups (*n* = 26 per group). Six participants did not complete the posttest because personal scheduling conflicts prevented attendance at the posttest session: two from each group. Thus, 72 participants completed both assessments and were included in the primary analysis (Table 1). Before testing, two instructors with badminton teaching or coaching experience evaluated participants’ high clears, drop shots, and basic footwork to verify that they possessed stable fundamental badminton skills but had not reached formal athlete level. The primary analyses included the 72 participants who completed both pretest and posttest; pretest data from participants who did not complete the posttest were retained only for overall data-quality summaries and were not included in the repeated-measures analyses.

**Table 1:** Baseline characteristics of participants included in the primary analysis.

Variable	Progressive temporal-occlusion group (*n* = 24)	Active-control group (*n* = 24)	Regular-activity control group (*n* = 24)
Age (years)	20.79 ± 1.96	21.00 ± 1.93	21.33 ± 1.79
Sex (male/female)	12/12	12/12	12/12
Years of badminton participation	3.37 ± 0.58	3.07 ± 0.64	3.38 ± 0.58
Weekly badminton participation (h)	3.81 ± 1.37	3.88 ± 1.20	3.91 ± 1.19
Dominant hand (right/left)	21/3	20/4	24/0
Test sequence (A → B/B → A)	12/12	12/12	12/12

Continuous variables are presented as mean ± SD; categorical variables are presented as counts. The table describes the 72 participants who completed both assessments and entered the primary analysis. At randomization, each group contained 26 participants.

### 2.2 Experimental design and stimulus materials

The experiment used a 3 (group: progressive temporal-occlusion, full-information active control, regular-activity control) × 2 (test session: pretest, posttest) × 4 (occlusion point: T0, T1, T2, T3) mixed design. Group was a between-participants factor, whereas test session and occlusion point were within-participant factors. T0, T1, T2, and T3 corresponded to 80 ms before racket–shuttle contact, 40 ms before contact, contact, and 40 ms after contact, respectively. Shot-direction anticipation accuracy was the primary performance measure and correct-response RT was the secondary measure. Pretest and posttest used two independent stimulus sets (A and B), and the A → B and B → A test sequences were counterbalanced within each group (12 participants per sequence). After pretest, participants were allocated 1:1:1 using sex-stratified variable-block randomization. Participants and training personnel were not blinded. Test presentation and response recording were automated, and data management and statistical analyses used anonymous participant codes.

Stimulus videos were recorded on a regulation badminton court. Four right-handed high-level badminton players performed forehand drop shots: two contributed only to training stimuli and two contributed only to test stimuli. Separating training and test performers reduced the likelihood that posttest performance reflected memory for performer-specific movement patterns.

A shuttle-feeding machine delivered high clears to the opposite side of the court. The camera was positioned on the feeding side, with the viewing direction arranged to approximate the receiver’s perspective. Video was recorded at 1920 × 1,080 pixels and 120 frames/s. Two coders independently identified, frame by frame, the first frame of racket–shuttle contact and defined it as time zero. Disagreements were resolved by joint frame-by-frame review. T0, T1, T2, and T3 were defined as 10 frames before contact, 5 frames before contact, the contact frame, and 5 frames after contact. Given the 120-frames/s sampling rate, these points corresponded to approximately −83.3, −41.7, 0, and +41.7 ms; for readability, they are referred to throughout the manuscript as −80, −40, 0, and +40 ms.

Stimulus sets A and B were independent, and each contained 240 base clips contributed equally by the two test performers. Within a single test, each base clip was assigned to only one occlusion condition and presented once; other occluded versions of that clip were not shown. Each occlusion point therefore comprised 60 unique clips. Stimuli were balanced across straight versus cross-court shots, left versus right landing direction, and performer. Landing direction was classified relative to the receiver’s perspective using the longitudinal center line of the receiving court; only shots whose first landing point clearly entered the designated target area were retained. Sets A and B were constructed using the same selection criteria and balancing rules to maximize comparability across stimulus sets and test performers. Training and test stimulus libraries were completely separate, and the number, repetition frequency, and rotation schedule of training clips were fixed in advance.

Testing took place in a quiet indoor setting with stable lighting. The display resolution was 1920 × 1,080 pixels at a fixed refresh rate of 240 Hz, and viewing distance was approximately 60 cm. Responses were entered on an external keyboard. The experiment was programmed in PsychoPy. The 120-frames/s videos were pre-decoded into frame sequences, with each video frame displayed for two monitor refresh cycles. Before data collection, a photodiode and high-speed camera were used to verify video offset, response-screen onset, and keypress timing so that the timing of critical events could be checked against the programmed schedule.

### 2.3 Test procedure and training conditions

Participants completed 8–10 practice trials before formal testing. Each test trial began with a 1,000-ms fixation cross, followed by the shot video. At the predetermined occlusion point, the video terminated and was immediately replaced by a gray response screen. Participants judged whether the shuttle would land on the left or right side and responded with the F or J key, with a maximum response window of 2000 ms. RT was measured from the onset of the response screen. No accuracy feedback was provided during pretest or posttest. Each test comprised 240 trials divided into four blocks; the four occlusion conditions were equally represented and randomly intermixed within each block, with short rest periods between blocks Figure 1.

1. Progressive temporal-occlusion group. Participants completed 8 weeks of training, with two sessions per week (16 sessions in total) and 50 trials per session. After each anticipation response, they received feedback indicating whether the response was correct and the correct shot direction. Training followed a fixed progression: both sessions in Week 1 used the +200-ms full-information condition; the two sessions in Week 2 used +160 and +120 ms, respectively; Weeks 3–7 used +80, +40, 0, −40, and −80 ms in sequence; and Week 8 used a mixed T0–T3 consolidation schedule (Table 2). Training and testing controlled video offset in full-frame units. At 120 frames/s, the nominal +200, +160, +120, +80, +40, 0, −40, and −80-ms conditions were implemented at 24, 19, 14, 10, 5, 0, −5, and −10 frames relative to contact, corresponding to approximately +200.0, +158.3, +116.7, +83.3, +41.7, 0, −41.7, and −83.3 ms. Nominal values are used throughout for readability. The training rationale was not simply to reduce video duration; it was to first establish a mapping between the hitter’s action and shot outcome under relatively complete information and then progressively move video offset earlier, thereby reducing post-contact shuttle-flight information and requiring judgments from earlier action information. The two video-training groups were matched for number of sessions, trials, and feedback exposures.
2. Full-information active-control group. The training period, number of sessions, number of trials, response requirements, and feedback format were identical to those of the progressive group. However, all 16 sessions used a fixed +200-ms post-contact full-information condition and did not include progressive occlusion. This offset retained relatively rich post-contact shuttle-flight information without directly showing the shuttle’s final landing point. The active-control condition therefore controlled for general practice factors including video exposure, repeated anticipation judgments, and outcome feedback. Importantly, the progressive group differed from the active-control group not only in the progressive sequencing of video offset but also in repeated exposure to earlier occlusion conditions. The design can therefore compare the overall effects of the two training programs, but it cannot isolate the independent contribution of progression itself from the contribution of practice under earlier occlusion.
3. Regular-activity control group. Participants maintained their usual badminton learning and extracurricular activity across the 8-week period and received no additional video-based perceptual training. All three groups recorded weekly badminton frequency, total participation time, and any additional sport-specific training to monitor badminton exposure outside the study. The immediate posttest was completed 48–72 h after the final training session.

**Figure 1:**
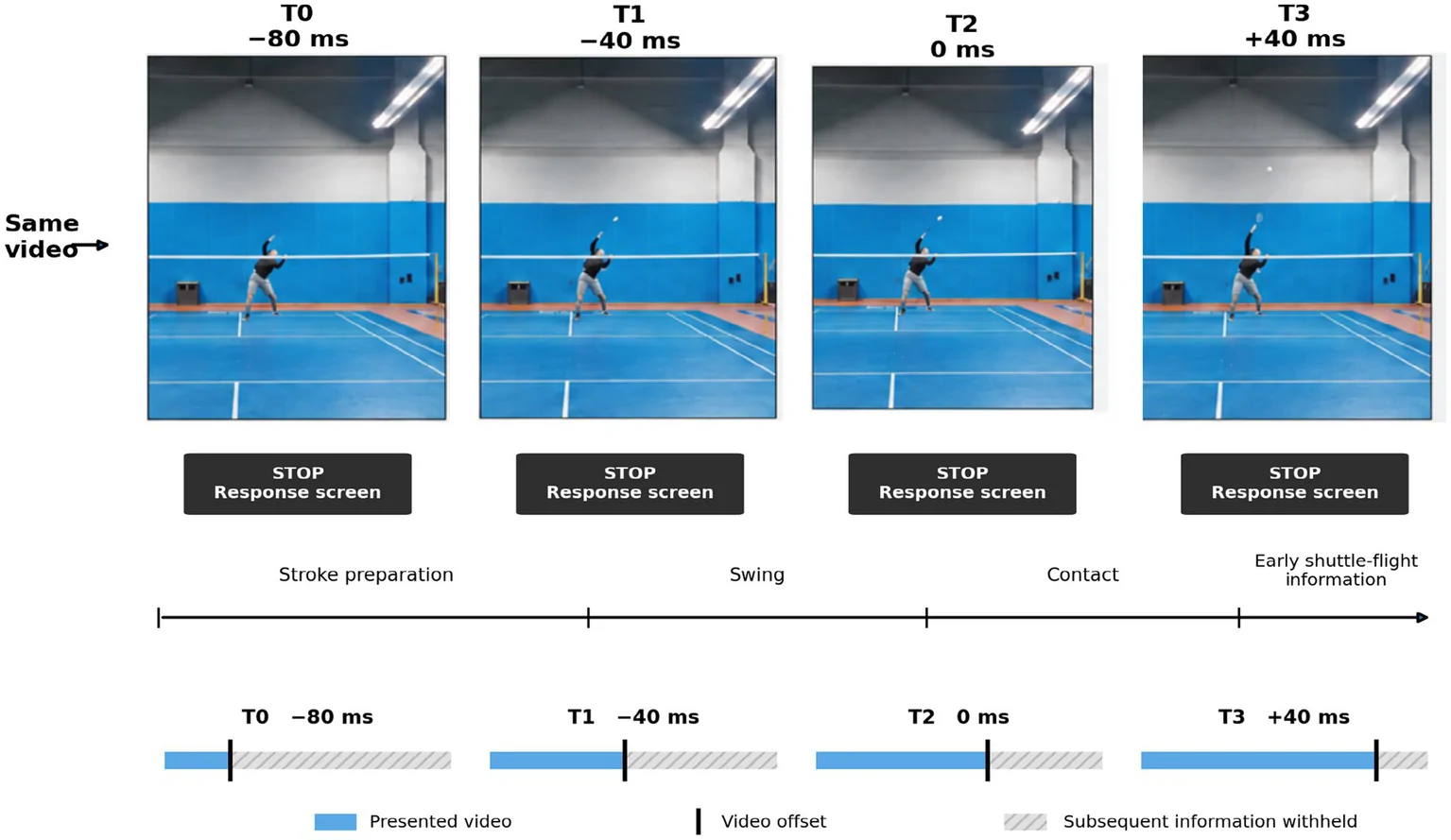
Temporal-occlusion procedure applied to the same base video at the four test points. The same base video is terminated at T0 (−80 ms), T1 (−40 ms), T2 (0 ms), or T3 (+40 ms). The presented segment ends at the black video-offset marker; subsequent information is withheld. The four on-court frames are drawn from the same base video.

**Table 2:** Eight-week training schedule.

Week	Sessions	Progressive temporal-occlusion group	Full-information active-control group
1	1–2	+200 ms full-information condition	+200 ms full-information condition
2	3–4	+160 → +120 ms	+200 ms full-information condition
3	5–6	+80 ms	+200 ms full-information condition
4	7–8	+40 ms	+200 ms full-information condition
5	9–10	0 ms	+200 ms full-information condition
6	11–12	−40 ms	+200 ms full-information condition
7	13–14	−80 ms	+200 ms full-information condition
8	15–16	Mixed −80/−40/0/+40 ms consolidation	+200 ms full-information condition

Both video-training groups completed 50 trials per session and received identical trial-by-trial outcome feedback. The active-control group remained at the +200-ms full-information offset throughout training.

### 2.4 Performance measures and statistical analysis

For the accuracy analysis, correct responses were coded as 1 and incorrect responses or failures to respond within 2000 ms were coded as 0. Only software failures, invalid keypresses, or other trials for which a response could not be classified were treated as missing. Correct-response RT included only trials with a correct judgment and a valid first keypress. Responses faster than 200 ms were excluded. Within each participant × test session × occlusion-point cell, RTs exceeding that cell’s mean ± 3 SD were classified as RT outliers and excluded from RT analyses. RT outlier screening did not alter the denominator used for accuracy.

Shot-direction anticipation accuracy was the primary performance measure and correct-response RT was the secondary performance measure. The primary comparison was between the progressive temporal-occlusion group and the active-control group, testing whether the progressive training program produced additional improvement under restricted shuttle-flight information when video exposure, anticipation practice, and feedback dose were matched. Comparisons across occlusion points were used to locate the temporal range of training-related benefits: T1 and T2 assessed performance with information available immediately before and around contact, T0 probed an earlier information boundary, and T3 served as a reference condition in which early shuttle-flight information was already available.

The 72 participants who completed both assessments were analyzed using a 3 (group) × 2 (test session) × 4 (occlusion point) mixed repeated-measures ANOVA. Greenhouse–Geisser correction was applied to effects involving occlusion point when sphericity was violated. Planned comparisons tested between-group differences in pretest-to-posttest change between the progressive and active-control groups at each occlusion point. Accuracy and correct-response RT each formed a family of four occlusion-point comparisons, and Holm adjustment was applied within each family. Holm’s step-down procedure controls the family-wise error rate while generally providing greater statistical power than the single-step Bonferroni correction; therefore, it was used for the planned comparisons in this study. In addition, with T3 as the reference, the mean training effect across T0–T2 was contrasted with the later-information condition to examine whether training benefits were relatively greater before shuttle-flight information was fully available. Ninety-five percent confidence intervals and corresponding effect sizes were reported. To assess robustness across analytic levels, trial-level accuracy was analyzed using a binomial generalized estimating equation (GEE) with a logit link, single-trial correct/incorrect response as the dependent variable, and participant as the clustering unit. Correct-response RT included valid correct trials only; RTs were log-transformed and analyzed using a Gaussian GEE with an identity link. Both GEE models included group, test session, occlusion point, and their interactions, used participant as the clustering unit, and employed robust standard errors.

All statistical tests were two-sided with *α* = 0.05. Because no drift-diffusion model was fitted, between-group training effects were interpreted only at the level of observable behavior and not as direct evidence of changes in latent evidence-accumulation or decision-boundary parameters.

## 3 Results

### 3.1 Sample and overall performance

All 78 participants completed randomization (26 per group). Six did not complete the posttest because of scheduling conflicts, two from each group, leaving 72 participants in the primary analysis. Training attendance was 97.14% in the progressive temporal-occlusion group and 97.66% in the active-control group. Baseline characteristics of participants included in the primary analysis are shown in Table 1. For participants who did not complete the posttest, pretest records were retained only for overall data-quality summaries and were not included in the primary repeated-measures analysis.

The pretest sample of 78 and posttest sample of 72 generated 36,000 raw trials. Using the pre-specified measure-specific cleaning rules, software failures, unclassifiable responses, and RT outliers were flagged separately for accuracy and RT analyses; RT outliers were not removed from the accuracy denominator. Among the 72 participants who completed both assessments, 23,796 correct trials with valid RTs were included in the correct-response RT analysis.

Pretest performance was broadly similar across groups. As video offset moved from T0 to T3, anticipation accuracy increased overall and correct-response RT decreased.

At posttest, accuracy in the progressive group was 61.53%, 71.63%, and 78.38% at T0, T1, and T2, respectively; corresponding values were 55.78%, 66.75%, and 74.63% in the active-control group and 53.40%, 61.97%, and 70.02% in the regular-activity control group. At T3, accuracy exceeded 90% in all three groups (Table 3; Figures 2, 3).

**Table 3:** Pretest and posttest anticipation performance across occlusion points.

Group	Occlusion point (ms)	Accuracy, pretest (%)	Accuracy, posttest (%)	RT, pretest (ms)	RT, posttest (ms)
Progressive temporal-occlusion group	−80	53.06 ± 6.17	61.53 ± 6.44	1437.06 ± 70.97	1225.84 ± 62.93
	−40	63.50 ± 7.71	71.63 ± 6.48	1351.88 ± 78.53	1144.71 ± 66.45
	0	66.73 ± 6.71	78.38 ± 5.91	1294.31 ± 65.95	1113.09 ± 75.81
	40	88.73 ± 4.24	91.58 ± 4.43	1168.71 ± 78.05	1089.80 ± 53.09
Full-information active-control group	−80	49.92 ± 6.38	55.78 ± 6.53	1439.34 ± 90.70	1307.87 ± 92.29
	−40	63.76 ± 7.75	66.75 ± 6.78	1353.45 ± 84.67	1223.43 ± 96.46
	0	68.66 ± 6.35	74.63 ± 6.03	1288.35 ± 104.26	1176.69 ± 93.69
	40	89.33 ± 4.20	90.81 ± 4.25	1166.24 ± 78.94	1109.52 ± 68.41
Regular-activity control group	−80	51.79 ± 5.62	53.40 ± 6.85	1428.67 ± 67.40	1374.48 ± 73.71
	−40	62.08 ± 6.35	61.97 ± 9.93	1341.46 ± 69.20	1313.90 ± 65.40
	0	65.81 ± 5.18	70.02 ± 6.02	1269.90 ± 68.05	1211.85 ± 74.03
	40	89.32 ± 3.53	90.17 ± 3.61	1151.98 ± 66.04	1126.28 ± 53.88

Values are mean ± SD. Correct-response RT is based only on trials with a correct judgment and a valid RT.

**Figure 2:**
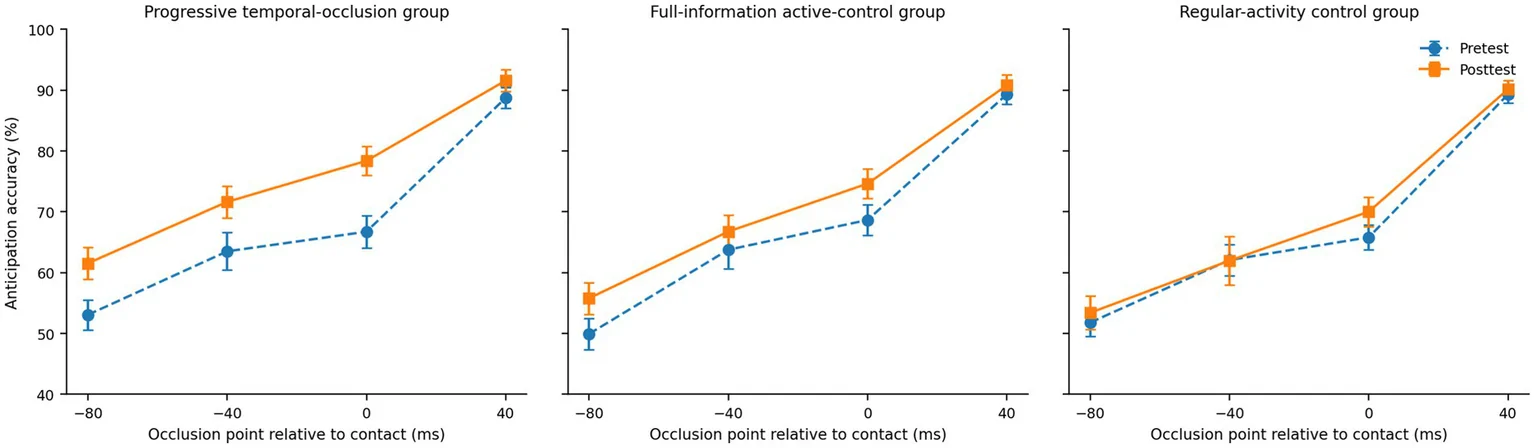
Pretest and posttest anticipation accuracy across occlusion points in the three groups. Error bars represent 95% confidence intervals.

**Figure 3:**
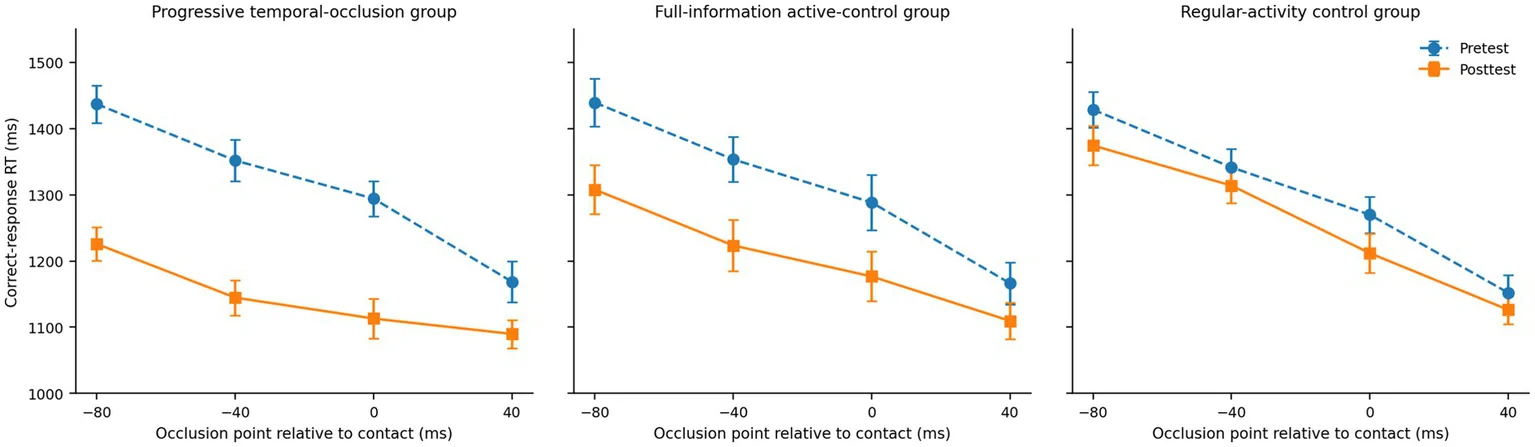
Pretest and posttest correct-response reaction time across occlusion points in the three groups. Error bars represent 95% confidence intervals.

### 3.2 Changes in shot-direction anticipation accuracy

Accuracy showed a significant group × test-session interaction [*F*₂,₆₉ = 17.83, *p* < 0.001, ηp^2^ = 0.34] and a significant test-session × occlusion-point interaction [*F*₂.₅₇,₁₇₇.₂₅ = 10.45, *p* < 0.001, ηp^2^ = 0.13]. The group × test-session × occlusion-point interaction was not significant [*F*₅.₁₄,₁₇₇.₂₅ = 1.52, *p* = 0.185, ηp^2^ = 0.04] (Table 4). Thus, overall evidence that the accuracy benefit differed systematically across information stages was limited, and the prespecified planned comparisons were interpreted accordingly.

**Table 4:** Mixed repeated-measures ANOVA for anticipation accuracy and correct-response RT.

Measure	Effect	Corrected df	*F*	*p*	GG ε	ηp^2^
Accuracy	Group	2, 69	8.84	<0.001	—	0.20
Accuracy	Test session	1, 69	113.15	<0.001	—	0.62
Accuracy	Occlusion point	2.50, 172.82	696.40	<0.001	0.835	0.91
Accuracy	Group × test session	2, 69	17.83	<0.001	—	0.34
Accuracy	Group × occlusion point	5.01, 172.82	2.14	0.063	0.835	0.06
Accuracy	Test session × occlusion point	2.57, 177.25	10.45	<0.001	0.856	0.13
Accuracy	Group × test session × occlusion point	5.14, 177.25	1.52	0.185	0.856	0.04
Correct-response RT	Group	2, 69	4.38	0.016	—	0.11
Correct-response RT	Test session	1, 69	161.71	<0.001	—	0.70
Correct-response RT	Occlusion point	2.89, 199.25	735.19	<0.001	0.963	0.91
Correct-response RT	Group × test session	2, 69	19.68	<0.001	—	0.36
Correct-response RT	Group × occlusion point	5.78, 199.25	6.52	<0.001	0.963	0.16
Correct-response RT	Test session × occlusion point	2.93, 202.02	37.89	<0.001	0.976	0.36
Correct-response RT	Group × test session × occlusion point	5.86, 202.02	7.87	<0.001	0.976	0.19

Effects involving occlusion point were Greenhouse–Geisser corrected where appropriate. GG ε = Greenhouse–Geisser epsilon.

Planned comparisons showed that, relative to the active-control group, the progressive group achieved a 5.14-percentage-point greater pretest-to-posttest increase in accuracy at T1 [95% CI: 1.01 to 9.27; Holm-adjusted *p* = 0.048] and a 5.68-percentage-point greater increase at T2 [95% CI: 2.16 to 9.20; Holm-adjusted *p* = 0.009]. Between-group change differences at T0 and T3 were 2.62 and 1.38 percentage points, respectively, and were not significant (Table 5). Because the omnibus three-way interaction was not significant, these planned contrasts indicate differences at prespecified time points but do not establish a stable occlusion-point-specific pattern in the accuracy training effect.

**Table 5:** Planned comparisons between the progressive temporal-occlusion and active-control groups.

Measure	Occlusion point (ms)	Progressive change	Active-control change	Between-group change difference	95% CI	Holm-adjusted *p*
Accuracy	−80	8.47	5.86	2.62	−0.54 to 5.77	0.204
Accuracy	−40	8.14	3.00	5.14	1.01 to 9.27	0.048
Accuracy	0	11.64	5.96	5.68	2.16 to 9.20	0.009
Accuracy	40	2.85	1.47	1.38	−0.73 to 3.50	0.204
Correct-response RT	−80	−211.22	−131.47	−79.75	−131.53 to −27.96	0.013
Correct-response RT	−40	−207.17	−130.02	−77.15	−131.21 to −23.08	0.013
Correct-response RT	0	−181.22	−111.66	−69.55	−115.96 to −23.15	0.013
Correct-response RT	40	−78.91	−56.72	−22.19	−65.11 to 20.74	0.304

Accuracy changes are expressed in percentage points; RT changes are in milliseconds. Negative RT values indicate faster responses. Between-group change difference = progressive temporal-occlusion change—active-control change.

A further stage contrast using T3 as the reference showed that, for the mean training effect across T0–T2, the progressive group exceeded the active-control group by 3.1 percentage points [95% CI: 0.55 to 5.64, *t*₄₆ = 2.45, *p* = 0.018, d = 0.71]. Relative to the regular-activity control group, the progressive group showed larger accuracy gains of 6.87, 8.24, and 7.43 percentage points at T0, T1, and T2, respectively, whereas the T3 difference was not significant. Differences in accuracy change between the active-control and regular-activity control groups did not reach Holm-adjusted significance at any of the four occlusion points.

### 3.3 Changes in correct-response reaction time

Correct-response RT showed a significant group × test-session interaction [*F*₂,₆₉ = 19.68, *p* < 0.001, ηp^2^ = 0.36] and a significant group × test-session × occlusion-point interaction [*F*₅.₈₆,₂₀₂.₀₂ = 7.87, *p* < 0.001, ηp^2^ = 0.19] (Table 4). Relative to the active-control group, the progressive group showed additional RT reductions of 79.75, 77.15, and 69.55 ms at T0, T1, and T2, respectively (all Holm-adjusted *p* = 0.013), whereas the difference at T3 was not significant (*p* = 0.304). The active-control group also showed larger RT reductions than the regular-activity control group at earlier occlusion points, indicating that repeated exposure to sport-specific actions, repeated direction judgments, and outcome feedback themselves produced a general practice benefit. The progressive group nevertheless showed additional improvement beyond this practice effect, without an evident speed–accuracy trade-off.

### 3.4 Sensitivity analyses

Trial-level GEE analysis of accuracy showed a significant group × test-session interaction (Wald *χ*^2^ = 6.89, *p* = 0.032), whereas the group × test-session × occlusion-point interaction was not significant (Wald *χ*^2^ = 6.91, *p* = 0.329), consistent with the aggregated-data analysis. In the trial-level log-RT GEE, the three-way interaction was significant (Wald *χ*^2^ = 43.98, *p* < 0.001), indicating that the occlusion-stage pattern in RT was robust across analytic levels.

## 4 Discussion

With the number of training sessions, number of trials, and feedback dose held constant, the progressive temporal-occlusion program produced additional gains in shot-direction anticipation relative to full-information video training. For accuracy, the progressive group improved more overall, and planned comparisons showed larger gains at T1 (−40 ms) and T2 (0 ms) than in the active-control group. However, the group × test-session × occlusion-point interaction was not significant, so these planned comparisons should not be taken as evidence of a stable occlusion-point-specific accuracy effect. In contrast, correct-response RT showed a significant three-way interaction, and the progressive group showed larger RT reductions across T0–T2, providing clearer statistical support for stage-dependent differences in RT.

The present findings are consistent with the broader literature showing that skilled performers generally outperform less-skilled performers in anticipation tasks, including when visual information is temporally restricted (Abernethy, 1988; Mann et al., 2007; Robertson et al., 2022). Early in the progressive program, relatively rich post-contact information allowed participants to repeatedly map the hitter’s action onto the resulting shot direction. As video offset moved earlier, later shuttle-flight information was progressively removed and judgments had to be completed with less available information. The additional accuracy gains at T1 and T2, together with the additional RT reductions across T0–T2, indicate that participants improved their direction-anticipation performance when information was restricted before and around contact. This behavioral change may reflect more effective use of action information available before video offset. However, visual-search behavior was not recorded and specific action cues were not selectively manipulated; the present data therefore do not establish whether training altered the extraction, selection, or integration of particular kinematic cues.

Sport anticipation does not rely on a single information source. With increasing sport experience, performers may use contextual priors and current kinematic information differently, and more skilled athletes often make better use of information that becomes available during the unfolding action (Chen et al., 2024; Wang D. et al., 2025). Information use also adapts when time is constrained. Lu et al. (2025) showed that behavioral benefits from congruent prior information were preserved under temporal pressure, accompanied by changes in EEG activity during preparatory and kinematic-information processing. Huang et al. (2026) likewise reported expertise-related differences in neural activity and functional connectivity during the processing of prior and kinematic information. Temporal-occlusion training research further shows that manipulating when action information becomes unavailable can improve visual anticipation in sport (Müller et al., 2024; Song et al., 2025). Badminton studies similarly indicate that sport-specific perceptual training can improve anticipation (Wang X. et al., 2025), while more skilled performers tend to show superior judgments before and around contact (Wu et al., 2026). The training effects observed here should therefore be interpreted as improved behavioral anticipation under early-information constraints, not as evidence that participants acquired perceptual or neural mechanisms equivalent to those of highly skilled athletes.

The pattern across occlusion points also shows that training benefits did not simply increase as video offset was moved earlier. At T0 (−80 ms), the action information available for judging direction was still limited. Although the progressive group improved at this point, it did not show a clear accuracy advantage over the active-control group. At T3, early shuttle-flight information was already available, accuracy exceeded 90% in all three groups, and between-group differences narrowed as shot direction became increasingly specified. Harris et al. (2022) proposed that anticipation integrates prior experience with current sensory evidence and adjusts the weighting of information sources according to their reliability. T1 and T2 occurred from 40 ms before contact to the moment of contact, when action information relevant to direction was increasingly available but post-contact shuttle-flight information had not yet fully emerged. Nevertheless, because the group × test-session × occlusion-point interaction for accuracy was not significant, the present data do not justify concluding that accuracy benefits were specifically concentrated within this interval.

Changes in correct-response RT provide complementary evidence. The active-control group also responded faster at earlier occlusion points after training, indicating a general practice benefit from repeated observation of sport-specific actions, repeated direction choices, and feedback. Even with this practice dose matched, the progressive group showed an additional RT reduction of approximately 70–80 ms at T0–T2, with no corresponding loss of accuracy and therefore no obvious speed–accuracy trade-off. The progressive program thus improved not only judgment performance but also the time required to complete the judgment and execute the keypress after information was terminated. This interpretation requires an important qualification: RT was measured from the onset of the response screen after video offset. It therefore reflects the total time required to complete the judgment and keypress after information became unavailable. It does not mean that participants formed their decision 70–80 ms earlier during video viewing, nor does it provide direct evidence that latent evidence-accumulation rate or decision boundaries changed.

The practice-matched active control and the separation of training and test stimuli strengthened interpretation of the training effects. The progressive and full-information groups completed the same number of video presentations, direction judgments, and feedback episodes, so the between-group differences cannot readily be attributed to training dose or feedback frequency alone. However, the progressive group differed from the active-control group in two linked respects: video offset was progressively advanced, and participants accumulated more practice under earlier occlusion conditions, whereas the active-control group remained at +200 ms. The present experiment therefore demonstrates an advantage of the progressive temporal-occlusion program as a whole over full-information training, but it cannot isolate whether that advantage arose from progressive sequencing, greater exposure to early occlusion, or their combination. Training and test performers were also independent, and posttest clips were not taken from the training set, reducing the likelihood that improvement reflected familiarity with performer-specific movement patterns. Existing work indicates that temporal-occlusion and other perceptual training can improve anticipation, but transfer across performers, tasks, and real competition is influenced by task representativeness and the degree of perception–action coupling (Brenton et al., 2019; Morris-Binelli et al., 2021; Dicks et al., 2009; Hadlow et al., 2018; Huesmann and Loffing, 2024).

From a training perspective, temporal-occlusion video practice may be a useful supplement to on-court badminton training. For learners with an established badminton foundation, full-information video practice can be supplemented with pre-contact and near-contact occlusion conditions that require directional judgments before the shuttle trajectory is fully revealed. Improvement in some measures within the active-control group shows that repeated viewing, judgment, and feedback alone can generate practice benefits; the additional gains observed in the progressive group suggest that anticipation practice under earlier information constraints may provide added value. Because transfer to an on-court task was not assessed, the present findings are best treated as guidance for structuring video-based anticipation practice rather than as evidence of improved match performance. The optimal progression schedule will require direct comparison of alternative training sequences.

This study has several limitations. First, the task used two-dimensional video and keypress responses and included only left-versus-right judgments of forehand drop shots; it therefore does not fully represent the perception–action coupling and multiple shot options present in real play. Second, no process measures such as eye tracking or cue-specific occlusion were included, so interpretations of training-related changes in information processing remain limited to behavioral performance. Third, only an immediate posttest was administered. Without delayed retention testing or an on-court transfer task, the durability of the training effects and their transfer to actual badminton performance remain unknown.

## 5 Conclusion

The 8-week progressive temporal-occlusion training program improved forehand drop-shot direction anticipation in university students with systematic badminton training experience on a video-based temporal-occlusion task. Compared with dose-matched full-information video training, the program produced additional improvements in selected anticipation measures. Planned comparisons showed larger accuracy gains at 40 ms before contact and at contact; however, because the three-way interaction for accuracy was not significant, these findings do not establish a clear occlusion-point-specific accuracy effect. Correct-response RT showed more consistent additional reductions from 80 ms before contact through contact.

## Data Availability

The raw data supporting the conclusions of this article will be made available by the authors, without undue reservation.
